# Protective effects of morin and propolis against cytarabine-induced neurotoxicity: a multi-biomarker approach

**DOI:** 10.1515/biol-2025-1289

**Published:** 2026-02-20

**Authors:** Hakan Bağ, Seval Yılmaz, Songül Çeribaşı

**Affiliations:** Biochemistry Department, Firat University Veterinary Faculty, 23200, Elazig, Türkiye; Pathology Department, Firat University Veterinary Faculty, 23200, Elazig, Türkiye

**Keywords:** cytarabine, neurotoxicity, morin, propolis, apoptosis

## Abstract

Cytarabine (Cyt) is a cornerstone chemotherapeutic agent for acute myeloid leukemia (AML) and various hematological malignancies. This study sought to investigate the neuroprotective potential of morin and propolis against Cyt-induced neurotoxicity. Forty-two Sprague Dawley rats were randomly assigned to six groups: control, morin (200 mg/kg/day), propolis (100 mg/kg/day), Cyt (100 mg/kg/day), morin + Cyt, and propolis + Cyt. Biochemical analysis of brain tissue revealed that Cyt administration significantly elevated malondialdehyde (MDA) levels and glutathione-S-transferase (GST) activity, while depleting catalase (CAT) and glutathione peroxidase (GSH-Px) activities. Immunohistochemical findings showed that Cyt increased 8-hydroxydeoxyguanosine (8-OHdG) and B-cell lymphoma/leukemia-2-associated X protein (Bax) expression, whereas it downregulated Glutathione peroxidase 4 (GPX4) and B-cell lymphoma/leukemia-2 (Bcl-2). Treatment with morin or propolis effectively reversed these oxidative and apoptotic markers, as evidenced by decreased MDA and Bax levels, alongside increased activities of antioxidant enzymes. Furthermore, the histopathological alterations induced by Cyt were markedly ameliorated by both antioxidants. These results suggest that Cyt-induced neuronal degeneration is driven by oxidative stress and apoptosis, processes that can be mitigated by morin and propolis supplementation.

## 1 Introduction

Cytarabine (Cyt; C_9_H_13_N_3_O_5_) constitutes an antineoplastic agent commonly employed in the treatment of acute myeloid leukemia (AML) and acute lymphocytic leukemia. However, due to its systemic toxicity, this agent has the capacity to cause tissue damage, particularly within organs such as the liver, kidneys, and brain, through mechanisms associated with oxidative stress [1], [2], [3]. The primary factors that preclude the oral administration of Cyt include its significant first-pass metabolism within the liver and the presence of the cytidine deaminase enzyme. Following intravenous administration, it exhibits a considerable volume of distribution owing to its low plasma protein binding capacity, a characteristic that facilitates its passage across the blood-brain barrier [4], [5], [6], [7]. The principal dose-limiting side effects associated with Cyt therapy include neurotoxicity, myelosuppression, gastrointestinal mucosal damage, keratoconjunctivitis, and oxidative stress. Neurotoxicity, in particular, represents a clinically significant complication that can impose considerable limitations on the treatment process. Cerebellar dysfunction resulting from Cyt manifests as motor coordination disorders, including ataxia, dysarthria, nystagmus, and gait disturbances. In assessments of pediatric patients with AML who were treated with high doses of Cyt, notable impairments in working memory were observed, despite the preservation of overall intelligence levels. These findings indicate that neurotoxic chemotherapeutic agents, such as Cyt, may be associated with cerebellar toxicity [8], [9], [10], [11], [12], [13], [14], [15], [16], [17]. In experimental studies, pronounced systemic and neurological effects have been reported in animal models treated with Cyt. These effects occur in a dose-dependent manner and include structural changes such as a reduction in the hepatosomatic index, decreases in body and brain weight, degeneration of Purkinje cells in the cerebellum, and cytotoxic damage in the granular cell layer [2], [18], [19], [20], [21], [22], [23], [24], [25]. In the pathophysiology of Cyt-induced neurotoxicity, increased production of reactive oxygen species (ROS) resulting from the inhibition of mitochondrial DNA synthesis and disruption of oxidative phosphorylation plays a central role. This state of oxidative stress triggers neurodegenerative processes and apoptosis by elevating levels of 8-hydroxyguanine (8-OHdG), which is formed through the reaction of ROS with guanine bases and serves as an important biomarker of cellular DNA damage [26]. Cyt, a pyrimidine nucleoside analog, induces neoplastic cell death by inhibiting the DNA polymerase enzyme through its active metabolite; however, simultaneously, it suppresses antioxidant enzyme activities, thereby disrupting cellular redox homeostasis in healthy neural tissue and contributing to cerebellar dysfunction [6], 27], 28]. Glutathione peroxidase 4 (GPX4) is a selenium-containing enzyme that plays a central role in protecting cells against oxidative stress and in the regulation of ferroptosis [29]. GPX4 reduces hydroperoxides and prevents ferroptosis; conversely, a decrease or inhibition of GPX4 activity initiates ferroptosis. GPX4 is a fundamental protective enzyme against neurotoxicity mediated by oxidative stress and ferroptosis in neurons. A reduction in GPX4 levels or impairment of its function exacerbates neurotoxicity [30], 31].

B-cell lymphoma/leukemia-2-associated X protein (Bax) is a member of the Bcl-2 family of proteins and generally plays a role in apoptotic pathways. Bax is an important protein examined during induction chemotherapy and in the assessment of apoptosis in patients with acute myeloid leukemia. B-cell lymphoma/leukemia-2 (Bcl-2) is one of the pro-survival proteins of the cell and supports the survival of cancer cells. Cyt triggers apoptosis and initiates cell death via the mitochondrial pathway. Bcl-2 inhibitors enhance this process and facilitate the death of cancer cells; however, activation of this mechanism in healthy tissues is an undesirable outcome [32], [33], [34].

There is no proven effective treatment option for Cyt-induced neurotoxicity other than supportive measures and discontinuation of the drug. This situation necessitates the investigation of new compounds that may reduce the toxic effects of Cyt.

Natural products have attracted significant interest due to their pleiotropic and health-promoting properties. These bioactive compounds are characterized by their ability to modulate multiple signaling pathways simultaneously and offer broad therapeutic potential against chemically induced toxicities and oxidative damage [35], 36]. Within the framework of these multifunctional natural agents, morin and propolis stand out for their potent antioxidant and cytoprotective capacities. Morin (3,5,7,2′,4′-pentahydroxyflavone) is a flavonol isolated as a yellow pigment from various plants, particularly those belonging to the Moraceae family. Studies have demonstrated that, in addition to its antidiabetic, anticancer, hepatoprotective, neuroprotective, anti-inflammatory, and antioxidant properties, morin is also a potent free radical scavenger [37], [38], [39], [40], [41]. The basis of these pharmacological effects lies in morin’s high antioxidant potential, which distinguishes it among polyphenols. Morin protects cellular components against oxidative damage by neutralizing free radicals. Structurally, this antioxidant activity depends on the presence of a double bond between the C2 and C3 atoms and on the electron-donating properties of the OH groups at the 2′ and 4′ positions of the B ring [42], 43]. Morin exerts its protective effect against neuronal damage by reducing oxidative stress, suppressing neuroinflammation, and downregulating proinflammatory mediators. Additionally, it has been shown to exhibit a cytoprotective effect against lipid peroxidation (LPO), thereby preserving cell membrane integrity and significantly reducing intracellular ROS production [44], [45], [46], [47], [48].

Propolis, also known as bee glue, is a natural bee product containing numerous phytochemicals. It is a resinous substance produced by honeybees (*Apis mellifera*) by mixing their saliva, which contains specific enzymes and beeswax, with exudates collected mainly from leaves and flower buds, stems, and the bark cracks of numerous tree species [49], [50], [51]. Propolis contains numerous organic compounds, including lipids, beeswax, essential oils, flavonoids, phenolic compounds, polyphenols, terpenes, terpenoids, coumarins, steroids, amino acids, and aromatic acids [52]. Propolis possesses a broad spectrum of biological and therapeutic properties, including antioxidant, antidiabetic, anti-inflammatory, anticancer, neuroprotective, hepatoprotective, immunomodulatory, and immunoinflammatory agents [50], 53], 54]. The mechanism underlying the neuroprotective properties of propolis is attributed to its ability to scavenge free radicals [55], 56]. However, the protective effects of these two potent antioxidants against Cyt-induced cerebellar damage and the underlying cellular mechanisms of this protection have not yet been comprehensively investigated. The aim of this study is to evaluate the potential protective effects of morin and propolis, known for their natural antioxidant properties, on Cyt-induced neurotoxicity, particularly through antioxidant, anti-apoptotic, and ferroptosis mechanisms.

## 2 Materials and methods

### 2.1 Drugs and chemicals

Morin hydrate, BLD pharma, Cas No: 654055-01-3. Propolis samples were collected from Elazığ province (Eastern Anatolia). Hand-collected propolis samples were stored in the dark and dried until processing. Cyt, KocakFarma^®^ (Korabin). Other chemicals were obtained from Merck (Darmstadt, Germany). Cyt, morin, and propolis doses were determined according to previous studies [6], 20], [57], [58], [59], [60], [61], [62].

### 2.2 Animals

The study used 42 male Sprague Dawley rats aged 2.5 months. The rats were obtained from the Fırat University Experimental Research Center (Elazığ/Turkey). Experiments were conducted under standard rat housing and feeding conditions (24 ± 3 °C, 12 h light/dark). The rats were provided with *ad libitum* tap water and pellet feed throughout the study.

**Ethical approval:** The research related to animal use has been complied with all the relevant national regulations and institutional policies for the care and use of animals, and has been approved by the Fırat University Animal Experiments Local Ethics Committee (Protocol No: 2024/16-04).

### 2.3 Experimental design

**Figure 1:**
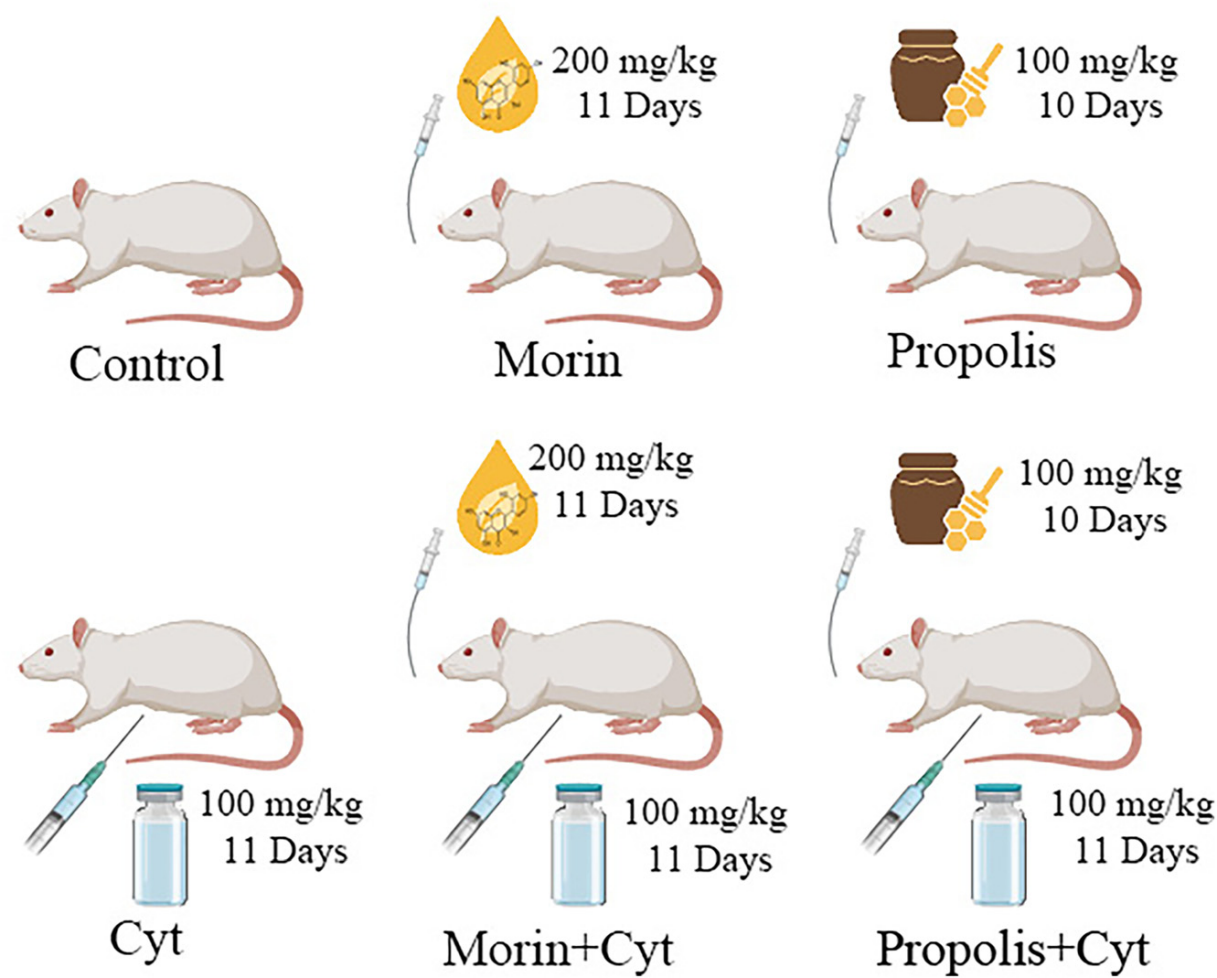
Schematic representation of the experimental design and treatment protocols. The study utilized 42 male Sprague Dawley rats divided into six groups (*n* = 7 each). The diagram illustrates the dosage, administration routes (oral gavage and IP injection), and the duration of treatments for morin, propolis, and Cyt.

The study consisted of 6 different groups, each containing 7 male Sprague Dawley rats. Rats were selected randomly. The groups were formed as follows (Figure 1). Group I (control): No treatment was administered to the control group. Group II (morin): Rats were administered morin at a dose of 200 mg/kg/day for 11 days via gavage. Group III (propolis): Rats were administered propolis at a dose of 100 mg/kg/day for 10 days via gavage. Group IV (Cyt): Cyt was administered intraperitoneally (IP) at a dose of 100 mg/kg/day for 11 days. Group V (morin + Cyt): Cyt was administered IP at a dose of 100 mg/kg/day for 11 days, 1 h after the administration of morin at a dose of 200 mg/kg/day. Group VI (propolis + Cyt): Rats received Cyt at a dose of 100 mg/kg/day for 10 days, administered 1 h after propolis administration at a dose of 100 mg/kg/day.

The rats were euthanized by decapitation without chemical anesthesia at the end of the specified days and periods.

### 2.4 Propolis extraction and biochemical examination

The propolis samples used in the study were collected from the Elazığ region. Raw propolis was prepared by dissolving it in 40 % ethanol. The detailed chemical composition of this propolis group was characterized by GC-MS analysis previously performed by Seven et al. [63] on a 70 % ethanol extract. The GC-MS analysis was performed using an Agilent GC 6890 chromatograph and an Agilent MSD 5973 mass detector. The main components of the propolis sample are presented in Table 1.

**Table 1:** Chemical composition of propolis extract determined by GC-MS analysis.

Component group	Content	TIC (%)^a^
Flavonoids	Chrysin (5.33), acacetin (3.02), naringenin (2.67)	11.02
Alcohol, terpenes, and quinones	Farnesol (20.64), 1-propen-1-thiol (4.51), 1-cyklohexene-1-methanol (4.64)	31.61
Aromatic acids	Palmitoleic acid (0.51), ferulic acid (0.43), cinnamic acid (0.41)	1.35
Aliphatic acids	Undecanoic acid (0.79), butanedioic acid (0.77), tetradecanoic acid (0.40)	2.64
Esters	4,3 acetyloxycaffeate (0.52), caffeic acid TMS ester (0.39)	0.91
Others	1H-cyclopentafuran (3.17), 3-hexane (1.61), 1,3 bis 5 propylbenzene (0.55)	5.35

^a^TIC%: total ion current percentage.

Morin was dissolved in 10 % dimethyl sulfoxide. At the end of the experiment, the animals were sacrificed and brain tissue was collected. The brain tissue was homogenized by diluting it with 1/10 distilled water and centrifuged at 3,500 rpm for 15 min. MDA levels and CAT, GSH-Px, and GST activities were examined in the brain tissue samples. MDA was determined spectrophotometrically according to the method developed by Placer et al. [64]. This method is based on the reaction between MDA, one of the products of LPO, and thiobarbituric acid (TBA). CAT enzyme activity was measured using the Aebi [65] method. The rate of hydrogen peroxide (H_2_O_2_) decomposition by the CAT enzyme was determined spectrophotometrically at 240 nm. GSH-Px activity was measured according to the Beutler [66] method, which is determined by spectrophotometrically reading the absorbance difference at 340 nm during the oxidation of NADPH to NADP. GST enzyme activity was measured using the Habig et al. [67] method. GST activity was determined spectrophotometrically by measuring the absorbance at 340 nm of the product formed by the conjugation of glutathione (GSH) and 1,2-dichloro-4-nitrobenzene.

### 2.5 Histopathological examination

Brain tissue samples obtained for histopathological examination were fixed in 10 % buffered formalin for 48 h. After rinsing the tissues overnight in tap water, they were processed through routine alcohol and xylene series, and paraffin blocks were prepared. Sections 5–6 µm thick were cut from the paraffin blocks, routinely stained with hematoxylin-eosin (HE), and examined under a light microscope [68].

### 2.6 Immunohistochemical analysis

Immunohistochemical analyses were performed using the Avidin-Biotin-Peroxidase method to evaluate the effects of morin and propolis on apoptosis and oxidative stress in the cerebral cortex. Immunohistochemical staining was performed using Bax antibodies to determine apoptotic cell death, Bcl-2 antibodies to evaluate the anti-apoptotic effects of morin and propolis, 8-Hydroxydeoxyguanosine (8-OHdG) antibodies to determine the severity of oxidative stress, and GPX4 antibodies to evaluate the effects of morin and propolis on ferroptosis. Five-micron-thick sections taken from paraffin blocks were deparaffinized with xylene and then dehydrated using a series of graded alcohols. To reveal antigenic receptors, sections were incubated in 0.01 M sodium citrate for 20 min, then washed with PBS and incubated in 3 % hydrogen peroxide prepared with methyl alcohol for 10 min to block endogenous peroxidase activity. To block nonspecific binding, the sections were incubated for 1 h in 1 % normal goat serum. The sections were then diluted with PBS according to the manufacturer’s procedure and stained with Bax (Invitrogen, Cas No: PA5-85918), Bcl-2 (Invitrogen, Cas No: PA5-27094), 8-OHdG (Bioss Company, Cas No: bs-1278R), and Glutathione Peroxidase 4 (GPX4) (Invitrogen, Cas No: PA5-102521) primary antibodies at 4 °C overnight. The sections were washed again in PBS, and biotinylated secondary antibody was added dropwise, and the sections were incubated for 10 min. The tissues were then washed with PBS and treated with Streptavidin peroxidase for 10 min. DAB (3,3′-Diaminobenzidine) was used as the color developing substrate. After applying this solution to the sections, the reaction was stopped as soon as color development began. Finally, the sections were stained with Mayer’s Hematoxylin for 2 min, passed through alcohol-xylol series, covered with a coverslip, and examined and evaluated under a light microscope at appropriate magnification.

### 2.7 Immunohistochemical evaluation

#### 2.7.1 Bax, Bcl-2 positivity in brain cortex

Following immunostaining, images of 10 randomly selected microscopic fields at 40× magnification in the cerebral cortex were captured using a digital camera. Cells showing Bax and Bcl-2 immunopositivity were counted using the Image J software (Image J 1.54p, Wayne Rasband and Contributors, National Institutes of Health, USA) [69].

#### 2.7.2 8-OHdG, GPX4 immunostaining intensity measurement in brain cortex

Images were acquired from 10 different areas of the cerebral cortex at 40× magnification using a digital camera. The intensity of 8-OHdG and GPX4 immunostaining was determined using the Image J program (Image J 1.54p, Wayne Rasband and Contributors, National Institutes of Health, USA) [70].

### 2.8 Statistical analysis

The normality of the data obtained from the study was analyzed using the Shapiro-Wilk test, and the homogeneity of variances was analyzed using the Levene test. For parameters with homogeneity of variance (*p* > 0.05), One-Way ANOVA was performed for intergroup comparisons, followed by Tukey’s post-hoc test. For parameters where variance homogeneity could not be established (*p* < 0.05), Welch ANOVA followed by Dunnett’s T3 post-hoc test was preferred. Data are presented as mean ± standard error of the mean (SEM), and *p* < 0.05 was considered statistically significant. All statistical analyses were performed using the SPSS software package (Version 22.0, IBM, SPSS, Armonk, USA).

## 3 Results

### 3.1 Biochemical results

**Figure 2:**
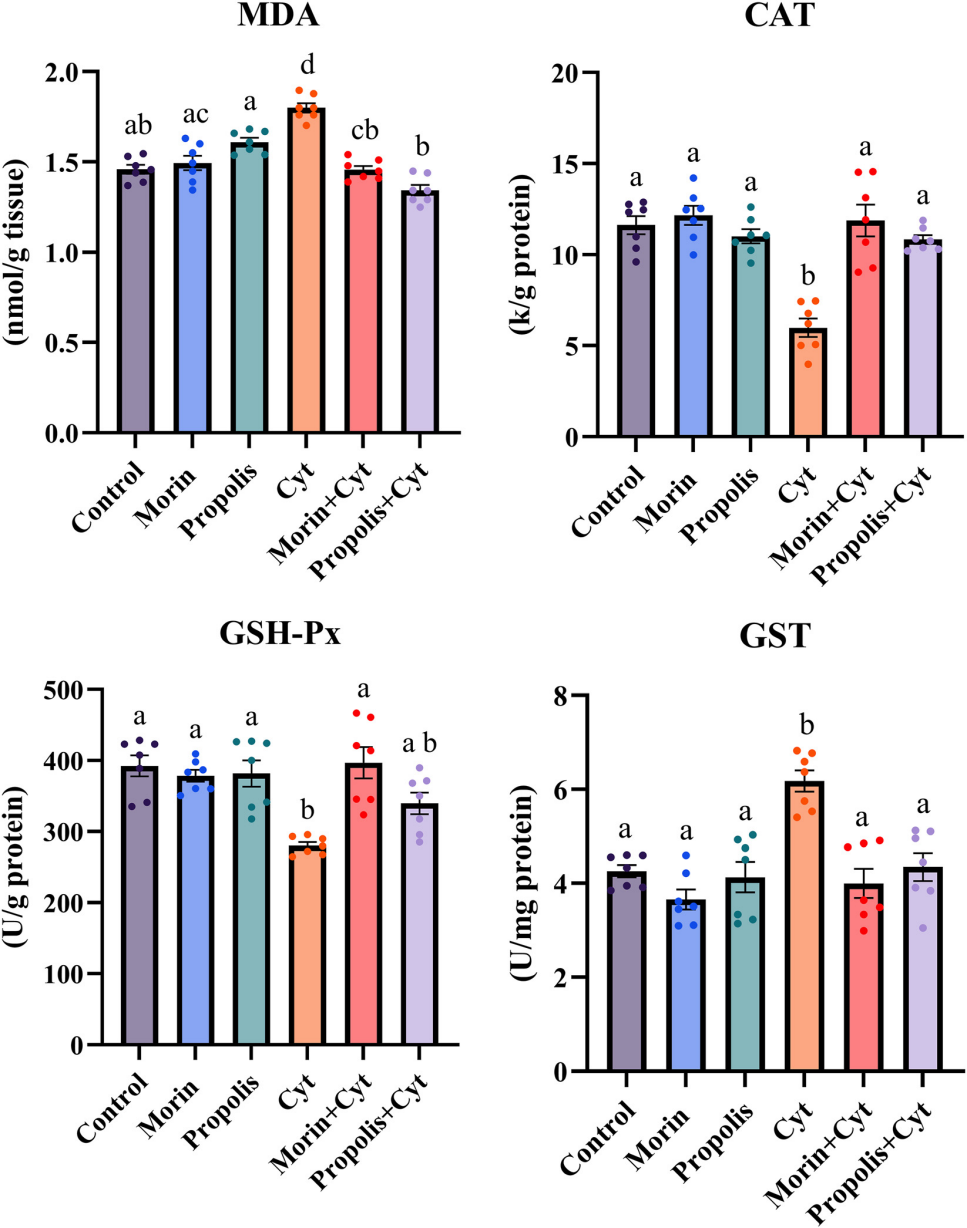
Brain tissue MDA level, as well as CAT, GST, and GSH-Px activities, were evaluated in the control and treatment groups (propolis, morin, Cyt, morin + Cyt, and propolis + Cyt). The normality of the data distribution was analyzed using the Shapiro-Wilk test, and the homogeneity of variances was analyzed using the Levene test. One-way ANOVA and Tukey post-hoc test were used for the parameter with homogeneous variance (MDA), while Welch ANOVA and Dunnett’s T3 post-hoc test were used for parameters with non-homogeneous variance (CAT, GSH-Px, GST). Results are presented as mean ± SEM. Overall *p* values for MDA, CAT, GSH-Px, and GST were determined as *p* < 0.0001. Groups with different superscript letters (a, b, c, d) are significantly different from one another (*p* < 0.05).

Figure 2 shows the MDA levels and CAT, GSH-Px, and GST antioxidant enzyme activities in the brain tissue of the control, morin, propolis, Cyt, morin + Cyt, and propolis + Cyt experimental groups. When the groups treated with morin and propolis alone were compared to the control group, no statistically significant difference was found in MDA levels and CAT, GSH-Px, and GST antioxidant enzyme activities (*p* ≥ 0.05).

Compared to the control group, the Cyt-treated group showed statistically significant increases in MDA levels (*p* < 0.0001) and GST activity (*p* = 0.0003), and statistically significant decreases in CAT activity (*p* < 0.0001) and GSH-Px activity (*p* = 0.002). When comparing the Cyt-treated group with the Morin + Cyt group, a decrease in MDA (*p* < 0.001) levels and GST (*p* = 0.002) activity, and a statistically significant increase in CAT (*p* = 0.002) and GSH-Px (*p* = 0.01) activity were observed. When comparing the Cyt-treated group with the propolis + Cyt group, an increase was observed in MDA (*p* < 0.0001) levels and GST (*p* = 0.006) activity, while a statistically significant decrease was observed in CAT (*p* = 0.0003) activity; however, no significant difference was observed in GSH-Px activity (*p* ≥ 0.05).

### 3.2 Histopathological results

**Figure 3:**
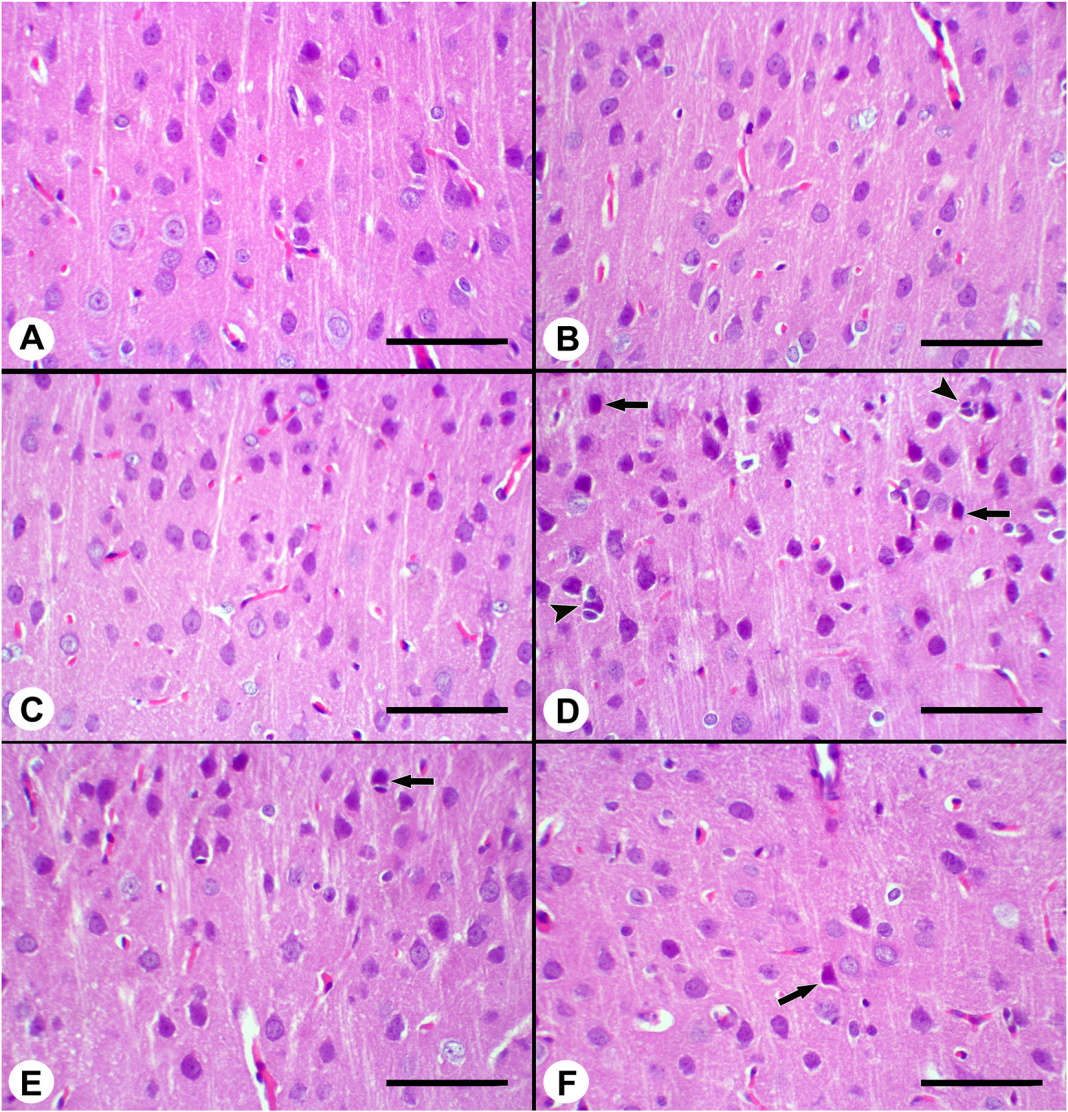
Histopathological changes in the brain cortex across experimental groups. (A) Normal appearance of brain cortex in control group, (B) normal appearance of brain cortex in morin group, (C) appearance of propolis brain cortex, (D) degenerative neurons with dark cytoplasm (arrows) and satellitosis (arrowheads) in brain cortex in Cyt group, (E) degenerative neuron with dark cytoplasm (arrow) in brain cortex in morin + Cyt group, (F) degenerative neuron with dark cytoplasm (arrow) in brain cortex in propolis + Cyt group, HE × 200, Bar = 100.

In the control, morin, and propolis groups, the brain cortex was observed to have a normal histological appearance. In the brains belonging to the Cyt group, neuronal degeneration was the most prominent change. Degenerative neurons were characterized by cytoplasmic shrinkage and pyknotic nuclei. Satellitosis, characterized by clusters of oligodendrocytes around degenerated neurons, was noted. In the morin and propolis groups combined with Cyt, the severity of the aforementioned lesions was found to be reduced compared to the Cyt group (Figure 3).

### 3.3 Immunohistochemical analysis

Data on the intensity of immunostaining with 8-OHdG and GPX4 antibodies using Image J and the number of positive cells in Bax and Bcl-2 immunostaining in control and experimental group rats are summarized in Table 2.

**Table 2:** Immunohistochemical staining scores for 8-OHdG and GPX4 and mean positive cell counts for Bax and Bcl-2 in the cerebral cortex of control and experimental groups.

	Control	Morin	Propolis	Cyt	Morin + Cyt	Propolis + Cyt	SH	*p*
8-OHdG	7.18 ± 0.14^a^	7.37 ± 0.16^a^	7.44 ± 0.05^a^	10.15 ± 0.38^b^	8.81 ± 0.39^c^	8.11 ± 0.21^ac^	0.13	*p* < 0.001
GPX4	14.30 ± 0.15^a^	14.35 ± 0.08^a^	14.53 ± 0.16^a^	11.94 ± 0.44^b^	13.21 ± 0.25^c^	13.16 ± 0.18^c^	0.12	*p* < 0.001
Bax	16.37 ± 1.50^ab^	15.10 ± 1.36^b^	14.63 ± 1.18^b^	26.03 ± 2.13^c^	20.13 ± 1.18^a^	18.13 ± 1.25^ab^	0.66	*p* < 0.001
Bcl-2	19.23 ± 1.43^a^	21.23 ± 1.59^a^	19.67 ± 1.45^a^	8.77 ± 0.54^c^	12.00 ± 0.79 ^bc^	12.93 ± 1.27^b^	0.61	*p* < 0.001

Results are presented as mean ± SEM. Statistical analysis between groups was performed using One-Way ANOVA followed by Tukey post-hoc test. Different superscript letters (a, b, c) indicate significant differences between groups (*p* < 0.05).

The highest intensity of 8-OHdG immunostaining, performed to evaluate free radical formation in the cerebral cortex, was observed in the Cyt group (Table 2). 8-OHdG immunostaining was observed in the neuronal nucleus and cytoplasm. The mildest 8-OHdG immunopositivity was detected in the control, morin, and propolis groups. It is noteworthy that the groups treated with Cyt plus morin and Cyt plus propolis reduced the intensity of 8-OHdG positivity compared to the group treated with Cyt alone (*p* < 0.001). The 8-OHdG positivity was similar in the groups treated with Cyt plus morin and Cyt plus propolis, and no statistical difference was determined between these groups (*p* ≥ 0.05).

**Figure 4:**
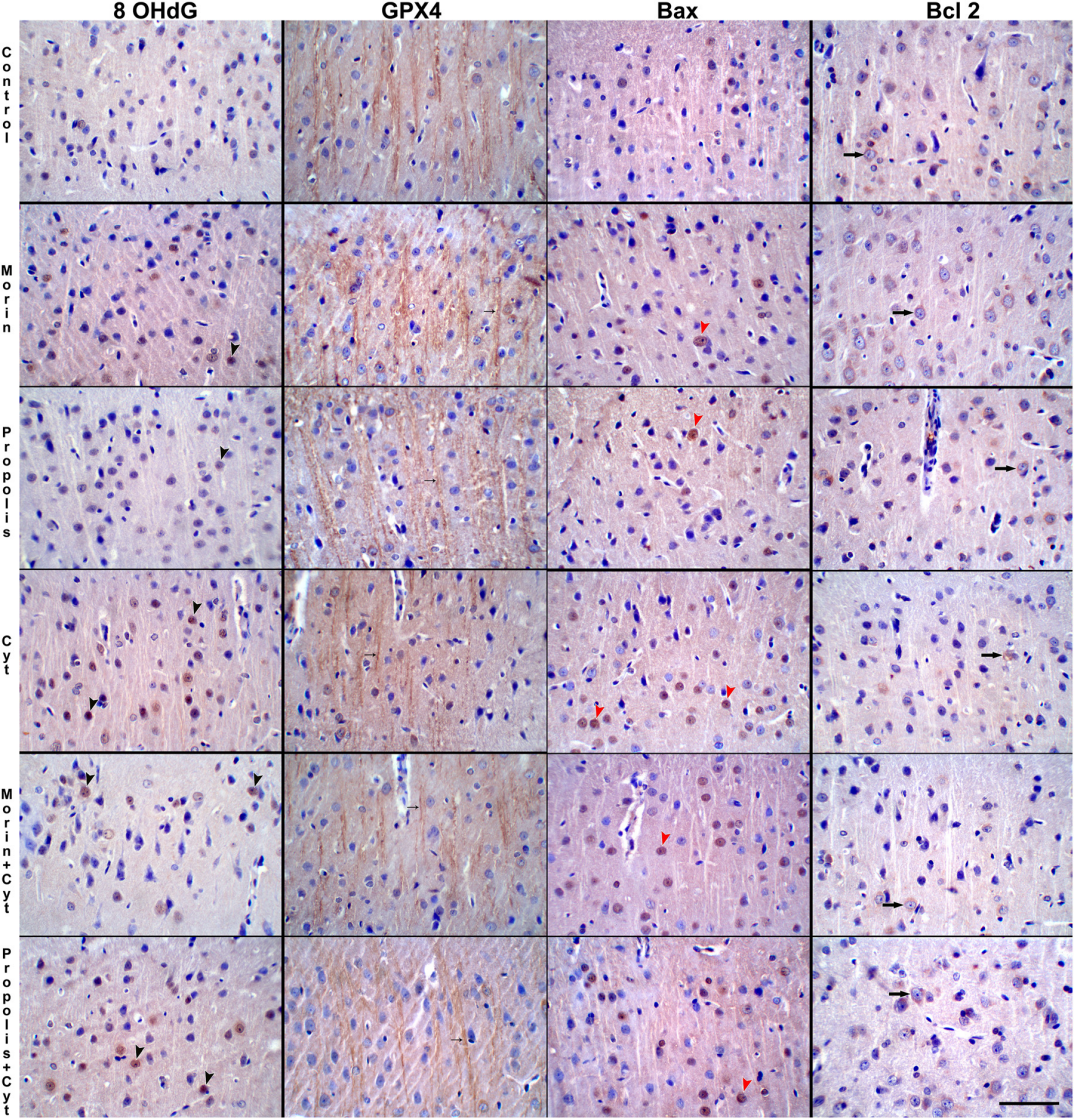
8-OHdG positivity in neurons (black arrowheads), GPX4 positivity in axonal neurofilaments (small arrows), Bax (red arrowheads) and Bcl-2 (large arrows) positivity in neurons in brain cortex in control and experimental groups MH × 200, Bar = 100 µ.

GPX4 immunopositivity, evaluated to assess ferroptosis in the cerebral cortex, drew attention in neurons in all groups in perinuclear and axonal neurofilaments. GPX4 immunopositivity was observed to be of similar intensity in the control, morin, and propolis groups and to continue uninterrupted longitudinally in axonal neurofilaments (Figure 4). In the Cyt-treated group, GPX4 positivity in neurofilaments in the cerebral cortex was found to be intermittent and short. The combination of Cyt with morin and propolis was found to cause a statistically significant increase in the intensity of GPX4 positivity compared to the Cyt group (*p* < 0.001).

Nuclear positivity was detected in Bax immunostaining performed to determine the number of apoptotic neurons in the cerebral cortex. The highest average number of Bax-positive cells was found in the Cyt group. Groups treated with Cyt together with morin and propolis significantly reduced neuronal apoptosis compared to the Cyt group (*p* < 0.001). The lowest number of apoptotic cells was found in the control, morin, and propolis groups. No statistical difference was found between these groups (*p* ≥ 0.05).

Bcl-2 immunostaining in the cerebral cortex was predominantly cytoplasmic, with a mild nuclear distribution. The groups with the highest number of Bcl-2 positive cells were the control, morin, and propolis groups, and no difference was found between these groups (*p* ≥ 0.05). The Cyt group had the lowest number of Bcl-2 positive cells. In the group treated with Cyt and propolis, an increase in Bcl-2 release in neurons was observed compared to the Cyt group (*p* < 0.001). In the group treated with Cyt and morin, an increase in Bcl-2 release in neurons was observed compared to the Cyt group, but this increase was not statistically significant (*p* ≥ 0.05).

## 4 Discussion

Cyt is a neoplastic agent frequently used in the treatment of acute myeloid and lymphocytic leukemia. However, its toxic side effects limit its therapeutic use [6], 71]. Cyt has a dose-limiting side effect that can cause neurotoxicity, especially when administered at high doses [72]. Cyt can cause serious complications, including mild drowsiness and imbalance, seizures, cerebral dysfunction, acute cerebellar syndrome, and even death, among its side effects on the central nervous system [73].

Studies have shown that exposure to sublethal levels of Cyt causes an increase in ROS production in dorsal root ganglion neurons [10], [73], [74], [75]. This toxicity, which arises due to oxidative stress in healthy tissues, poses an obstacle to increasing chemotherapy doses [76], 77]. Oxidative stress, apoptosis, and inflammation are thought to play a role, but the cause of Cyt related toxicity is not fully understood.

Cyt is a chemotherapeutic agent with antimetabolite properties, primarily exerting its effect by inhibiting DNA replication and thereby arresting cell division. However, it is known that Cyt may also be effective on non-proliferative cells and, in particular, may increase ROS production by disrupting mitochondrial functions. Increased ROS levels lead to a marked oxidative stress response in brain tissue, where the antioxidant defense system is limited. In this study, MDA levels and antioxidant enzyme activities were measured to determine the oxidative stress caused by Cyt in brain tissue and to identify the regulatory effect of morin and propolis, which are natural antioxidants. Cyt has been reported to cause ROS in brain tissue. Cyt-induced ROS production triggers the neurotoxicity mechanism by disrupting cellular homeostasis and causing oxidative damage to DNA, protein, and lipid structures [25], 78], 79]. MDA is a peroxidation product of polyunsaturated fatty acids and is scientifically accepted as an indicator of oxidative stress [80], 81]. Particularly due to Cyt, the plasma membrane is the first target of LPO, and MDA formation stands out as an indicator of Cyt-related oxidative stress [20], 25], 82]. Brain tissue contains a high amount of lipids and is therefore sensitive to LPO. This leads to an increase in MDA levels. According to the results obtained, Cyt significantly increased MDA levels in rat brain tissue. On the other hand, there was a decrease in MDA levels with morin and propolis application (*p* < 0.001). Studies have also reported that Cyt application increases MDA levels [2], 25], [79], [80], [81], [82], [83], [84], [85]. Patel et al. [20] reported that Cyt administered at a dose of 200 mg/kg increased MDA levels when compared to lower doses of 50 and 100 mg/kg. Similar results were obtained in our study after Cyt administration. Reactive species arising from the reaction of Cyt-derived free radicals with oxygen or H_2_O_2_ interaction may increase LPO. The LPO process is closely related to neurotoxicity, which causes cell death due to oxidative stress resulting primarily from a shift in the intracellular prooxidant-antioxidant ratio in favor of prooxidants. If cellular antioxidant defense systems fail to keep ROS levels below a dangerous threshold, oxidative stress occurs. This excessive ROS production may result from antioxidant defense failure or both [86], 87].

The deactivation and removal of ROS depends on the activity of antioxidant defense systems, including SOD, CAT, and GSH-Px [87], [88], [89]. Antioxidant enzymes are considered the primary defense system that protects biological macromolecules from oxidative damage. The decrease in GSH-Px activity due to Cyt is closely related to the significant increase in ROS production, which induces severe oxidative stress and consequently depletes or suppresses cellular antioxidant defense mechanisms [2], 84]. Low GSH-Px activity suggests that Cyt causes toxicity through mitochondrial dysfunction and oxidative stress.

CAT is one of the key enzymes involved in reducing oxidative stress by converting ROS compounds such as H_2_O_2_ into water and oxygen. ROS derived from Cyt can overwhelm the cellular antioxidant capacity, leading to depletion or inactivation of the CAT enzyme. Furthermore, oxidative stress can potentially downregulate the synthesis of CAT and other antioxidant enzymes by triggering signaling pathways that regulate gene expression [90], 91]. In our study, consistent with findings in the literature, Cyt administration was shown to significantly reduce CAT enzyme activity in brain tissue [1], 2], 84]. Brain tissue is particularly susceptible to oxidative damage due to its high oxygen consumption, weak endogenous antioxidant capacity, and lipid-rich structure. Therefore, changes in CAT activity can be considered a biochemical indicator of neurotoxic processes. Studies show that Cyt can trigger oxidative damage in neuronal structures and initiate neuroinflammatory processes. The decrease in CAT activity may be an important factor facilitating these processes. This situation may contribute to clinical conditions such as chemotherapy-induced cognitive impairment (chemo-brain) [92]. The above results suggest that morin and propolis may provide protection against Cyt-induced oxidative stress by reducing MDA levels and increasing antioxidant enzyme activities compared to the Cyt-treated group. One study reported that morin prevented the increase in ROS and MDA levels in gentamicin-treated rats, caused an increase in GSH levels, and regulated SOD and CAT enzyme activities [93]. Another study reported beneficial effects on these antioxidant enzyme activities in ammonium chloride-treated rats [94]. These studies support the results obtained in our study. GST is an important detoxification enzyme that protects cells against toxic substances and oxidative stress. It uses GSH to render harmful compounds less toxic. The GST enzyme plays a role in the defense mechanism against Cyt, and an increase in GST activity has been detected. This indicates an increased need to detoxify the electrophilic byproducts of Cyt. This finding can be linked to the transition process to apoptosis reported by Guzmán et al. [95].

Researchers are working on various approaches, such as the use of antioxidants and modulation of oxidative stress pathways, to minimize damage to non-cancerous tissues during chemotherapy [76], 96], 97]. In this context, our study evaluated the neuroprotective potential of morin and propolis against Cyt.

Morin has demonstrated neuroprotective effects in oxidative stress-induced neurotoxicity models. It has been reported to improve neuronal function and reduce neuroinflammation [46], 47]. The anticancer effects of morin have been extensively studied and it has been found to inhibit cell proliferation in many tumors, including leukemia, squamous cell carcinoma, colorectal cancer, and breast cancer [41], 98]. Morin treatment has been observed to reduce MDA levels, and these results are consistent with previous studies [99], [100], [101]. Increases in CAT and GSH-Px activity support the antioxidant effects of morin, while the decrease in GST activity indicates that morin interacts directly with reactive compounds [99], 100], [102], [103], [104], [105], [106], [107].

Propolis is notable as a compound rich in phenolic acids, flavonoids, and natural antioxidants. Propolis has significant potential in terms of biologically active components such as phenolic acids and flavonoids. The limitations of current chemotherapy methods, such as high costs and toxic effects, necessitate research into safer and more effective alternative compounds [108], [109], [110]. Previous studies have reported that propolis reduces oxidative stress in treated groups [111], 112]. It is known that the bioactive compounds in propolis can stimulate the expression and synthesis of CAT in cells. Furthermore, propolis can increase the stability and activity of the CAT enzyme, leading to enhanced enzymatic function. By increasing CAT activity, propolis helps improve the cellular antioxidant defense system. This allows these cells to cope better with oxidative stress and maintain their normal functions. Propolis has been reported to improve immune function by reducing oxidative damage, alleviate inflammation, and provide protection against chronic diseases associated with oxidative stress, primarily cardiovascular diseases, neurodegenerative disorders, and certain types of cancer [61], 113], 114]. In our study, it was found that CAT activity increased significantly in the groups treated with propolis.

In our study, consistent with previous studies, a significant decrease in MDA levels was observed in the groups treated with propolis.

In our study, the fact that no significant difference was found between the Cyt and Cyt + propolis groups in terms of GSH-Px activity suggests that the antioxidant effect of propolis may have enzyme-specific limitations. The antioxidant capacity of propolis largely depends on its direct free radical scavenging effect and the regulation of certain antioxidant enzymes (e.g., SOD and CAT) and may not have shown the same level of effect on GSH-Px activity. This may be primarily related to the dose and duration of application. The increase in GSH-Px activity may generally be closely related to glutathione metabolism, selenium availability, and gene expression. It is possible that the applied propolis dose or duration was insufficient to activate this specific enzyme system. Furthermore, it is conceivable that the effect of propolis may be more pronounced in the early phases of oxidative stress, but that the GSH-Px response was limited at the time of measurement.

Studies have observed that propolis reduces GST activity [115], 116]. Various phenolic compounds found in propolis may directly affect the GST enzyme. It is thought that GST activity decreases as a result of the interaction of these compounds with GST [117].

The study revealed that Cyt application caused significant neurotoxic effects in the rat brain cortex using histopathological and immunohistochemical methods. The histopathological findings observed in the Cyt group, such as neuronal degeneration, pyknotic nuclei, cytoplasmic shrinkage, and satellitosis, indicate that the neurotoxic effect of Cyt causes serious structural damage in the cerebral cortex. These findings are consistent with studies indicating the toxic effects of Cyt on the central nervous system [20], 21]. In contrast, it has been observed that the application of morin and propolis, either alone or in combination with Cyt, reduces the severity of these lesions. This demonstrates the neuroprotective potential of both compounds.

The observation of similar immunohistochemical and histopathological profiles in the control, morin, and propolis groups indicates that these compounds do not have toxic effects on their own and may be a reliable treatment option.

Histopathological analyses confirm neurotoxicity in the Cyt group with findings such as marked neuronal degeneration, pyknotic nuclei, and satellitocytosis. Immunohistochemical analyses support that oxidative stress, ferroptosis, and apoptosis induced by Cyt are modulated by morin and propolis. The intense positivity observed in the Cyt group in 8-OHdG immunostaining confirms that free radical formation and DNA damage are one of the fundamental mechanisms of neurotoxicity [16]. The significant reduction in 8-OHdG positivity by morin and propolis indicates the antioxidant properties of these compounds. In particular, it can be said that the radical scavenging effects of morin and propolis, derived from their phenolic structures, alleviate oxidative stress-induced neuronal damage [16], 115], 118].

GPX4 immunoreactivity is weak in the Cyt group, indicating that the ferroptotic process is active. Ferroptosis is a cell death mechanism characterized by LPO-related neuronal membrane damage. Studies clearly show that Cyt application triggers this process. In contrast, morin and propolis treatments are thought to inhibit LPO by increasing GPX4 expression, thereby preventing ferroptosis [119], 120]. This effect may be related to the bioactive components of propolis and the anti-ferroptotic properties of morin.

During the apoptotic process, Bax opens membrane pores while Bcl-2 prevents the opening of mitochondrial membrane pores, thereby exerting an anti-apoptotic effect [121], 122]. The high number of apoptotic neurons in the Cyt group in Bax immunostaining indicates that Cyt activates pro-apoptotic pathways [123]. The high Bax positivity in the Cyt group reveals the pro-apoptotic effects of the drug; morin and propolis, on the other hand, exhibited apoptosis-inhibitory properties by reducing Bax expression [123], 124]. Increased Bcl-2 positivity in the morin and propolis groups indicates that these compounds support cellular survival mechanisms. The regulation of the Bax/Bcl-2 ratio suggests that morin and propolis regulate apoptosis via the intrinsic pathway. The reduction in the number of Bax-positive cells following morin and propolis administration demonstrates that these compounds inhibit apoptosis [124], 125].

Bcl-2 is one of the key anti-apoptotic proteins that plays a critical role in suppressing apoptosis via the mitochondrial pathway. In Bcl-2 immunostaining, decreased positivity in the Cyt group indicates weakened anti-apoptotic defense. In the study, the balancing of the Bax/Bcl-2 ratio by Cyt, morin, and propolis applications supports that their neuroprotective effects are dependent on apoptosis regulation. It also suggests that Bcl-2 supports neuronal survival by regulating the intrinsic pathway of apoptosis. Bcl-2 is one of the key anti-apoptotic proteins that plays a critical role in suppressing apoptosis via the mitochondrial pathway. The direct regulatory effect of propolis on cell survival via the mitochondrial apoptotic pathway by increasing Bcl-2 expression is based on the possibility that biologically active components such as flavonoids, phenolic acids, and caffeic acid in propolis may increase the transcription of anti-apoptotic genes [126], 127]. It is known that the antioxidant and cytoprotective effects of morin may arise mainly through the reduction of oxidative stress, modulation of intracellular signaling pathways, or suppression of pro-apoptotic factors [128], 129]. In this study, it was determined that Bcl-2 expression decreased by 54.4 % in the Cyt-treated group compared to the control group. In contrast, Bcl-2 expression increased by 36.8 % in the morin-treated group and by 47.4 % in the propolis-treated group compared to the Cyt group. The results obtained are consistent with the literature.

The similar protective effects of morin and propolis suggest that these substances act through antioxidant and anti-apoptotic pathways. Propolis exhibits potent antioxidant effects due to the flavonoids, phenolic compounds, and other biologically active substances it contains, while morin’s potential to reduce ROS production and protect mitochondrial function has also been supported in this study. Histopathological evaluations also supported the biochemical findings, revealing that tissue integrity was better preserved in the groups treated with morin and propolis.

In this study, morin and propolis were evaluated separately to clearly demonstrate their specific effects on oxidative stress and ferroptosis pathways. Our results show that both have potent neuroprotective roles individually, while their combined application may provide greater protection through synergistic interactions. Such synergy could further reduce systemic toxicity by allowing the use of lower doses of chemotherapeutic agents. Investigating these combined effects remains an important goal for our future studies.

## 5 Conclusions

Currently, there is no established treatment to reduce Cyt-induced neurotoxicity and supportive measures are generally taken and the use of the drug is discontinued. Therefore, the development of new treatment approaches that can mitigate the harmful effects of Cyt on the nervous system is a major necessity. The results obtained from this study reveal that morin and propolis offer a protective potential against Cyt-induced cerebellar damage at the preclinical level. However, more comprehensive research in clinically relevant models and human studies is needed before these findings can be recommended as a supportive strategy in cancer patients.

## 6 Limitation

The current study has some limitations. First, only male rats were used to eliminate potential variability caused by hormonal cycles. While this allowed for a more focused examination of biochemical pathways, the absence of female subjects limits the generalizability of our findings. Future studies involving both sexes are needed to fully understand the effects of cyt neurotoxicity on different biological profiles. Secondly, the absence of a positive control group treated with a clinically accepted neuroprotective agent is another limitation. While such a group would allow for a comparative analysis of the efficacy of morin and propolis, this study primarily focused on the dose-dependent effects and mechanistic pathways of these natural bioactives. Finally, although our findings show that morin and propolis reduce Cyt-induced oxidative stress and apoptosis, the direct interactions of these compounds with specific intracellular signaling pathways, such as Nrf2/HO-1 and p53, have not yet been fully elucidated. The lack of direct molecular evidence obtained by methods such as Western blot or RT-qPCR is a mechanistic limitation.

Future studies examining these molecular markers will provide a more comprehensive understanding of therapeutic targets.
